# Improved Draft Genome Sequence of *Bacillus* sp. Strain YF23, Which Has Plant Growth-Promoting Activity

**DOI:** 10.1128/MRA.00099-19

**Published:** 2019-04-11

**Authors:** Ye Xia, Seth DeBolt, Qin Ma, Adam McDermaid, Cankun Wang, Nicole Shapiro, Tanja Woyke, Nikos C. Kyrpides

**Affiliations:** a Department of Plant Pathology, The Ohio State University, Columbus, Ohio, USA; b Department of Horticulture, University of Kentucky, Lexington, Kentucky, USA; c Department of Biomedical Informatics, The Ohio State University, Columbus, Ohio, USA; d Department of Agronomy, Horticulture, and Plant Science, South Dakota State University, Brookings, South Dakota, USA; e Department of Energy Joint Genome Institute, Walnut Creek, California, USA; University of Maryland School of Medicine

## Abstract

We report here the improved draft genome sequence of Bacillus sp. strain YF23, a bacterium originally isolated from switchgrass (Panicum virgatum) plants and shown to exhibit plant growth-promoting activity. The genome comprised 5.82 Mbp, containing 5,933 genes, with 193 as RNA genes, and a GC content of 35.10%.

## ANNOUNCEMENT

Bacillus is a genus of Gram-positive and rod-shaped bacteria in the phylum *Firmicutes*. Bacillus spp. generally produce endospores, which can help the bacteria survive under stress conditions, such as extreme temperature, or under terrestrial soil conditions, which experience periodic drought stress (1–3). Some strains of Bacillus have been reported to promote the growth of different plants through diverse mechanisms (4–6). Bacillus sp. strain YF23 originated from switchgrass (Panicum virgatum L. var. Alamo) plants, one of the most important biofuel crops (7). Bacillus sp. YF23 was isolated from the endophytic compartment of switchgrass, which was growing on a reclaimed coal-mining site in western Kentucky. This bacterium showed significant growth-promoting activity on greenhouse-propagated switchgrass plants, indicating its potential to benefit the host plant under certain conditions and increase the yield and/or fitness of the biofuel crop (7). The aim of this study was to generate the genome of Bacillus sp. YF23 found in the endophytic compartment, as this may provide clues into its metabolic features and mechanisms for host interaction.

The switchgrass plants were collected from a coal-mining site in Kentucky (7, 8). Then, the shoots and roots of the switchgrass plants were cut into 3- to 5-cm segments and were sterilized with 20 to 30% Clorox bleach for 15 min to kill the surface-localized microbes. The segments were washed with the sterilized water 3 to 5 times. Further, the plant samples were cut into 1- to 1.5-cm segments and put on the plates with the tryptic soy agar medium (Sigma, USA). The plates were incubated in an incubator with a constant temperature of 26°C for 3 to 5 days. Bacterial strains from different tissues were isolated and further purified by growing them on the tryptic soy agar medium plates 2 to 3 times. One of the isolates, Bacillus sp. YF23, was then obtained and further purified (7, 8). For DNA extraction, Bacillus sp. YF23 was first cultured in the tryptic soy broth medium (Sigma) and grown on a shaker at room temperature for 1 to 2 days. Then, the broth containing bacterial cells was centrifuged, and the cell pellets were used for DNA extraction. The genomic DNA was extracted by using the cetyltrimethylammonium bromide (CTAB) approach developed by the Department of Energy Joint Genome Institute (DOE-JGI [9]). The genomic DNA was sequenced at the DOE-JGI using Pacific Biosciences (PacBio) technology. The PacBio SMRTbell library was constructed and sequenced with 86× depth (10).

**FIG 1:**
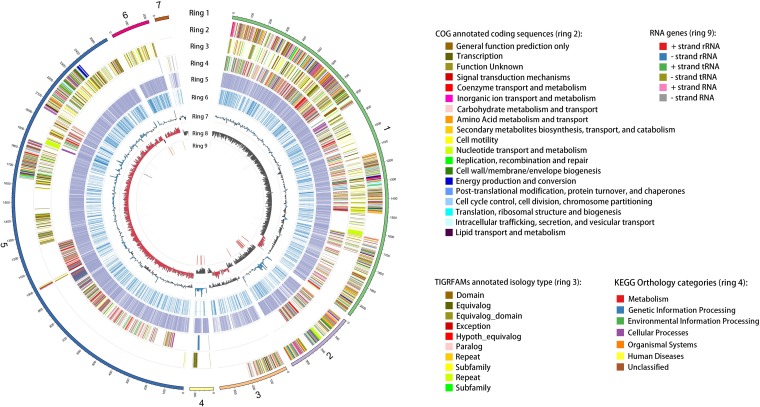
Circular representation of the *Bacillus* sp. YF23 genome generated using the Circos software. Features include the following: ring 1, 7 contigs of genome sequences; ring 2, Clusters of Orthologous Groups (COG)-annotated coding sequences; ring 3, TIGRFAM-annotated coding sequences; ring 4, KEGG orthology regions; ring 5, Pfam-annotated genes; ring 6, transmembrane helix regions; ring 7, GC content, with blue indicating above and black indicating below the genome average of 35.1%, with a 5-kb window; ring 8, GC skew, with red indicating above and black indicating below zero, with a 5-kb window; ring 9, RNA genes.

A total of 5,820,595 genome sequence reads were generated for Bacillus sp. YF23, yielding an assembly of 7 contigs (Fig. 1), by using Circos software analysis with the default settings (11). The code in its entirety, including specific parameters and settings, used to generate Fig. 1 can be found in a GitHub repository (see https://github.com/Wang-Cankun/Bacillus-sp.-YF23-Circos-scripts). The average read length for raw reads of >5 kb was 7,849 bp. Reads were assembled, quality controlled, and error corrected using HGAP version 2.3.0 with the default settings (12). The scaffold *N*_50_ value is 2 Mb. The genome annotation was carried out using the JGI Integrated Microbial Genome (IMG) system (13). Genes were identified using Prodigal 2.5 (14). The genome contains a total of 5,933 genes and has 35.10% GC content. The numbers of total protein-coding genes and protein-coding genes with predicted function are 5,740 and 4,670, respectively. The numbers of genes in biosynthetic clusters and genes coding signal peptides are 568 and 268, respectively. A total of 193 RNA genes were identified. Among them, 44 are rRNA genes, 116 are tRNA genes, and 33 are other RNA genes. For the rRNA genes, 14 are 5S rRNA, 14 are 16S rRNA, and 16 are 23S rRNA (Fig. 1).

The genome information provides insight into the functional mechanisms and application of this beneficial bacterium in enhancing switchgrass plant growth and health for biofuel production.

### Data availability.

The whole-genome sequence has been deposited at DDBJ/EMBL/GenBank under the accession no. PRJNA243950. The version described in this paper is the first version. The associated sequence data can also be found at the Joint Genome Institute (JGI) portal with the IMG taxon oid no. 2603880214 (https://genome.jgi.doe.gov/portal/BacillusspYF23_FD/BacillusspYF23_FD.info.html).
